# Monitoring patients on chronic treatment with antidepressants between 2003 and 2011: analysis of factors associated with compliance

**DOI:** 10.1186/s12889-015-2493-8

**Published:** 2015-11-26

**Authors:** M. Catalina Serna, Jordi Real, Inés Cruz, Leonardo Galván, Elisabet Martin

**Affiliations:** Universidad de Lleida- IRB-Lleida, Lleida, Spain; Centre d’Atenció Primària Eixample, Institut Català de la Salut, Lleida, Spain; Unitat de Suport a la Recerca Lleida-Pirineus, Àmbit Atenció Primària Lleida, Institut Universitari d’Investigació en Atenció Primària Jordi Gol (IDIAP Jordi Gol), Rambla Ferran, 44, 3ª planta, 25007 Lleida, Spain; Facultat de Medicina i Ciències de la Salut, Universitat Intenacional de Catalunya, Sant Cugat, Barcelona, Spain; Centre d’Atenció Primària Primer de Maig, Institut Català de la Salut, Lleida, Spain; Servei Català de la Salud, Lleida, Spain; Centre d’Atenció Primària Consell de Cent, Institut Català de la Salut, Barcelona, Spain

**Keywords:** Depression, Medication adherence, Antidepressant drugs

## Abstract

**Background:**

Clinical practice guidelines consider the use of antidepressants as one of the standard treatments for anxiety disorders, due to the significant improvements obtained in quality of life and functional disability. In addition, in patients who have not achieved a favorable response after 3 months of psychotherapy, antidepressants are recommended as part of a combined treatment approach. This combination with psychotropic drugs and psychotherapy appears to be indicated from baseline in patients with moderate, severe or recurrent depression. In the last decade, antidepressant prescription rates in general practice have increased between 4 and 10 times. Depression presents high rates of relapse and recurrence. Treatment is often interrupted prematurely, leading to increases in both relapse rates and health care costs. Few studies have analysed the chronic use of antidepressant drugs and long-term adherence. Objective: To evaluate compliance with antidepressant treatment between 2003 and 2011 and to explore the associated factors.

**Methods:**

Retrospective cohort study of antidepressant dispensing. Setting: Health Region of Lleida between 2003 and 2011. Participants: Patients with chronic prescription of antidepressants (ATC code NO6A) during 2003 were followed up until December 2011. The sample comprised 3684 subjects. Main measures: The compliance rate was calculated on the basis of the number of units withdrawn from the pharmacy and the theoretical number of units required according to the scheduled duration of treatment: compliance was defined in cases with scores greater than or equal to 80 %.

**Results:**

12.5 % of patients received chronic antidepressant treatment for at least 4 years. Mean age was 54 years, and 73.2 % of patients were female. Almost a third (32.4 %) presented anxiety disorders and 26.5 % mood disorders. The overall compliance rate was 22 % (28 % in patients with depression, and 21 % in patients with anxiety). According to gender, compliance rates were 21.4 % for males and 22.4 % for females. Compliance was more likely in patients with polypharmacy.

**Conclusions:**

One in 4 patients complied with treatment. Factors associated with better compliance were polypharmacy and diagnosis of depressive or mixed anxiety-depressive disorder.

## Background

After cardiovascular diseases, mental illness is the second leading cause of disease burden in economically developed countries, and one of the categories that most reduces the number of years of disease-free life [1]. Although the prevalence of depression has not increased over time [2], depressive disorders account for 40.5 % of the disability-adjusted life years caused by mental illness [3]. The global current prevalence of anxiety disorders is 7.3 %, ranging from 5.3 % in African cultures to 10.4 % in Euro/Anglo cultures [4]. The European ESEMeD epidemiological study found a lifetime prevalence of mood disorders of 14.7 % [5]. In Spain the prevalence of depressive episodes has been reported to be 10.5 %, making it the most common mental disorder [6].

Clinical practice guidelines vary from country to country. The NICE guidelines recommend psychological interventions as the first line of therapy and favour pharmacological treatment only as a later additional step in the case of non-response to psychotherapy. The use of antidepressants is one of the standard treatments for anxiety disorders, due to the significant improvements obtained in quality of life and in functional disability. Routine use of benzodiazepines should be avoided because they are associated with tolerance and dependence, and antipsychotics are associated with a number of adverse effects [7].

In patients with depression who have not achieved a favourable response after three months of psychotherapy, antidepressants are recommended as part of a combined treatment approach. This combination with psychotropic drugs and psychotherapy appears to be indicated from baseline in patients with moderate, severe or recurrent depression [8].

Depression presents high rates of relapse and recurrence [9]. After the first episode, the 2-year recurrence rate is 40 %, and after two episodes, the risk of recurrence at 5 years is 75 %. Furthermore, 10-30 % of patients do not recover completely; their symptoms persist, or they develop dysthymia [10]. In the last decade antidepressant prescription rates in general practice have increased between 4 and 10 times [11, 12]. The appropriateness of long-term treatment with antidepressants is currently being debated [13], although the rates of treatment compliance in chronic follow-up is unknown. Treatment is often interrupted prematurely, leading to increases in both relapse rates and health care costs [14]; some research shows that 40 % of patients discontinue the use of antidepressant medication during the first 3 months [15].

Few studies have analysed the relation between chronic use of antidepressant drugs and long-term compliance. The present study aimed to assess compliance with antidepressant treatment in a Spanish health region between 2003 to 2011 and to study the factors associated with compliance.

## Methods

### Data sources

A retrospective cohort study of the dispensing of antidepressants. Data were taken from prescriptions made in the Lleida Health Region between January 1, 2002 and December 31, 2011.

#### Data source

The data were obtained from the database of the Catalan Health Service for the dispensing of prescription drugs in pharmacies. In Catalonia, since the presentation of the health card is required to obtain medication with a Social Security prescription, users can be unambiguously identified. The prescription of antidepressants corresponds to group NO6A of the Anatomical Therapeutic Chemical classification in use in Spain [16] (Table 1).

**Table 1:** Prescriptions most frequently dispensed during the two study periods

	Period			
	2003–2007		2008–2011	
ATC Description	N°	(%)	N°	(%)
Amitriptyline	594	(16.12)	429	(11.64)
Clomipramine	324	(8.79)	215	(5.84)
Lofepramine	1	(0.03)	0	(0.00)
Mianserin	115	(3.12)	71	(1.93)
Oxitriptan	9	(0.24)	1	(0.03)
Imipramine	27	(0.73)	26	(0.71)
Minaprine	1	(0.03)	0	(0.00)
Nortriptyline	18	(0.49)	12	(0.33)
Tranylcypromine	2	(0.05)	0	(0.00)
Trazodone	124	(3.37)	144	(3.91)
Trimipramine	2	(0.05)	2	(0.05)
Fluoxetine	1173	(31.84)	529	(14.36)
Maprotiline	101	(2.74)	47	(1.28)
Doxepin	3	(0.08)	4	(0.11)
Fluvoxamine	96	(2.61)	36	(0.98)
Moclobemide	1	(0.03)	0	(0.00)
Venlafaxine	687	(18.65)	538	(14.60)
Sertraline	861	(23.37)	463	(12.57)
Dosulepin	3	(0.08)	0	(0.00)
Reboxetine	68	(1.85)	48	(1.30)
Mirtazapine	312	(8.47)	293	(7.95)
Bupropion	0	(0.00)	90	(2.44)
Citalopram	972	(26.38)	814	(22.10)
Paroxetine	1633	(44.33)	950	(25.79)
Escitalopram	740	(20.09)	984	(26.71)
Duloxetine	203	(5.51)	402	(10.91)
Agomelatine	0	(0.00)	88	(2.39)

Affiliation data (date of birth and gender), administrative status (includes death / change of address to another region) and medical diagnoses of anxiety and depression were extracted from the primary care information systems, in accordance with the International Classification of Diseases Version 10 (Diagnosis of anxiety: ICD-10 F40-F43; Diagnosis of depression: ICD-10: F30-F39).

#### Variables

The variables studied were: age at beginning of study, gender, units of antidepressants withdrawn from the pharmacy, polypharmacy, and clinical diagnosis justifying the prescription. A patient’s level of polypharmacy was defined as the number of different active principles prescribed monthly during the study period [17, 18]. Polypharmacy was divided into 4 groups, as follows (low: use of <2 drugs, normal: concomitant use of 2 to 2.7 drugs; moderate: 2.7 to 4.3 drugs, and high: > 4.3 drugs).

#### Selection of the cohort

Patients with a new prescription of an antidepressant during 2003 and still receiving treatment after January 2008 were followed up until December 2011. The sample comprised 3684 subjects (26.8 % males and 73.2 % females, mean age of 53.7 years (SD = 17.8).

#### Exclusion criteria

Patients who were dispensed antidepressants in 2002 or had received amitriptyline for neuropathic pain were excluded from the study (*n* = 317). 

#### Definition of compliance with treatment

Compliance with treatment was defined based on the units of medication dispensed during the treatment period. Treatment duration was calculated for each patient on the basis of the number of months from the first dispensing in 2003 to the last one before December 31, 2011. For each antidepressant, the number of units per month needed by the patient was considered according to the recommended daily dose defined by the WHO’s Centre for Drugs Statistics Methodology [19]. The compliance rate was thus calculated from the number of units dispensed at the pharmacy and the number of units theoretically required for the duration of episode. By consensus, compliance rates of 80 % until the end of treatment were considered adequate. A duration of at least six months of antidepressant treatment was also required [20]. Treatment was defined as chronic when a patient had received prescriptions on two or more occasions during the period.

#### Statistical analysis

A descriptive analysis was performed of the cohort considering frequencies and percentages. The compliance rate was estimated with a confidence interval of 95 % using normal approximation. To determine its possible association with other variables, the compliance rate was described and the Chi-square significance test was performed. Adjusted odds ratios (OR) of compliance with treatment were estimated by multivariate logistic regression models. The complete model and a second model with variables that showed statistical significance, two logistic models (Enter method) were adjusted. The Hosmer-Lemeshow test and area under the curve ROC (AUC) were computed to evaluate the performance of the multivariable models. P values less than 0.05 were considered statistically significant”.

## Results

During 2003, 29,517 individuals (8.6 % of the total registered population) were dispensed antidepressants in pharmacies in the Lleida Health Region. Of the patients who started antidepressant treatment in 2003, 12.5 % were still receiving chronic treatment after 4 years. The mean age of these patients was 54 years (SD: 17.82), and 73.2 % were women. In 2003, the general population in the Health Region comprised 309,786 inhabitants: 48.2 % were women and the mean age was 42 years (SD:23.9).

Anxiety disorders accounted for 25 %, mood disorders 19 %, mixed disorders 7 %, and in 49 % of cases no diagnosis was recorded.

The most frequently prescribed drug during the early period (years 2003–2008) was paroxetine followed by fluoxetine and sertraline. In the later period (2008–2011) paroxetine remained the most frequently prescribed, followed by escitalopram and then venlafaxine third (Table 3).

**Table 2:** Compliance with treatment stratified according to different variables

		Compliance		95 % CI para %	
Variable	N	n	(%)	(lower-upper)	*p* value
Global	3684	816	(22.1)	(20.8–23.5)	
Gender					
Male	988	211	(21.4)	(18.7–24.0)	0.483
Female	2696	605	(22.4)	(20.8–24.0)	
Age group					
Under 35	582	85	(14.6)	(11.7–17.5)	<0.001(*)
35–64	1901	411	(21.6)	(19.7–23.5)	
Over 65	1201	320	(26.6)	(24.1–29.2)	
Polypharmacy					
=<2	826	59	(7.1)	(5.4–8.9)	<0.001(*)
2–2.6	822	123	(15.0)	(12.5–17.5)	
2.7–4.3	1057	276	(26.1)	(23.4–28.8)	
>4.3	979	358	(36.6)	(33.5–39.6)	
Diagnosis recorded					
None	1785	351	(19.7)	(17.8–21.5)	<0.001(*)
Only anxiety	923	194	(21.0)	(18.3–23.7)	
Only depression	704	197	(28.0)	(24.6–31.4)	
Both	272	74	(27.2)	(21.8–32.6)	
Drug dispensed^a^					
Amitriptyline	787	167	(21.2)	(18.3–24.1)	0.479
Paroxetine	1880	332	(17.7)	(15.9–19.4)	<0.001(*)
Fluoxetine	1324	323	(24.4)	(22.0–26.8)	0.014(*)
Citalopram	1363	318	(23.3)	(21.0–25.6)	0.186
Sertraline	1024	266	(26.0)	(23.2–28.7)	0.001(*)
Venlafaxine	932	309	(33.2)	(30.1–36.2)	<0.001(*)
Clomipramine	414	142	(34.3)	(29.6–39.0)	<0.001(*)
Mirtazapine	492	153	(31.1)	(26.9–35.3)	<0.001(*)
Trazodone	230	45	(19.6)	(14.3–24.8)	0.33
Maprotiline	122	44	(36.1)	(27.4–44.8)	<0.001(*)
Imipramine	42	16	(38.1)	(23.1–53.1)	0.012(*)
Nortriptyline	25	10	(40.0)	(20.4–59.6)	0.031(*)
Fluvoxamine	118	39	(33.1)	(24.4–41.7)	0.004(*)

*Significance using chi-squared test: *p*-value <0.05

^a^Some of the active principles were not considered because patient numbers were insufficient

As regards the number of different drugs prescribed, 68.6 % of patients had received treatment with 1 or 2 antidepressants; 17.3 % with three, 7.8 % with four 4 % with five, and the remaining 2.3 % up to thirteen different antidepressant drugs.

The overall compliance was 22 % (95 % CI: 20.8 to 23.8 %). There were no statistically significant differences between genders: compliance rates were 21.1 % (*n* = 211) in males and 22.4 % (*n* = 605) in females. Compliance increased with age, rising to 26.6 % in the over-65 age group (*n* = 320). As regards polypharmacy, increasing the number of drugs also improved the compliance rate. Patients diagnosed with depression had a compliance rate of 28 % while in those diagnosed with anxiety the rate was 21 % (Table 2).

**Table 3:** Logistic regression models of the likelihood of better compliance with treatment

		Model 1^a^			Model 2^b^		
Variable			95 % CI for OR			95 % CI for OR	
Category		OR	(lower -upper)	*p*-value	OR	(lower-upper)	*p*-value
Sex							
Female		1.04	(0.85–1.26)	0.721			
Polypharmacy							
=<2 (Ref.)				<0.001			<0.001
2–2.6		2.11	(1.51–2.96)	<0.001	2.10	(1.51–2.93)	<0.001
2.7–4.3		4.67	(3.39–6.41)	<0.001	4.48	(3.30–6.10)	<0.001
>4.3		8.85	(6.33–12.35)	<0.001	8.20	(6.02–11.11)	<0.001
Age group							
Under 35 (Ref.)				0.389			
35–64		1.00	(0.75–1.33)	0.991			
Over 65		0.87	(0.63–1.20)	0.392			
Diagnosis recorded							
None (Ref.)				<0.001			<0.001
Only anxiety		1.41	(1.13–1.75)	0.002	1.45	(1.17–1.80)	<0.001
Only depression		1.58	(1.26–1.97)	<0.001	1.63	(1.31–2.03)	<0.001
Ambos		1.74	(1.25–2.42)	0.001	1.84	(1.33–2.54)	<0.001
Drug dispensed							
Amitriptyline		0.81	(0.66–1.00)	0.047			
Paroxetine		0.57	(0.48–0.68)	<0.001	0.57	(0.48–0.68)	<0.001
Fluoxetine		1.09	(0.91–1.30)	0.335			
Citalopram		1.05	(0.88–1.25)	0.604			
Sertraline		1.11	(0.93–1.34)	0.256			
Venlafaxine		1.92	(1.59–2.30)	<0.001	1.99	(1.66–2.39)	<0.001
Clomipramine		1.78	(1.40–2.27)	<0.001	1.80	(1.41–2.28)	<0.001
Mirtazapine		1.39	(1.10–1.75)	0.005	1.44	(1.14–1.81)	0.002
Trazodone		0.58	(0.40–0.83)	0.003	0.60	(0.42–0.85)	0.005
Maprotiline		1.61	(1.07–2.43)	0.023	1.64	(1.09–2.46)	0.018
Imipramine		1.70	(0.86–3.38)	0.129			
Nortriptyline		1.41	(0.56–3.52)	0.462			
Fluvoxamine		1.45	(0.94–2.24)	0.089			
Hosmer & Lemeshow test				0.003			0.781
Discrimination (Area Under Curve COR)		0.749		<0.001	0.729		<0.001

^a^Model 1: Multivariate association of compliance with all factors (the complete model)

^b^Model 2: Multivariate association of compliance with all statistically significant factors

Ref.: Reference category

At multivariate level, the factors associated with compliance were high levels of polypharmacy (>4.3), diagnosis of depressive or mixed disorder, and consumption of clomipramine, mirtazapine, maprotiline, or venlafaxine independently of gender and age. Furthermore, compliance with paroxetine and trazodone was lower after adjusting for age, gender and drugs dispensed (Table 3).

## Discussion

In 2003, a total of 29,517 (8.6 %) individuals were dispensed at least one antidepressant unit from pharmacies in the Lleida Health Region. Of the patients who started treatment with antidepressants in 2003, 12.5 % remained in treatment after 4 years. The mean age of patients was 54 years (SD: 17.82).

According to some reports, one-third of patients with depression present episodes or symptoms for more than 2 years [21]. Other patients present recurrences and require chronic medication.

In our population, anxiety disorders accounted for 25 %, mood disorders 19 %, mixed disorder in 7 %, and in 49 % no diagnosis was recorded. The descriptive approach based on the diagnostic criteria of the CIE-10 [22] and the DSM-IV [23] has improved the identification and treatment of mental disorders worldwide. However, even the creators of these systems recognize that their main achievement has been to improve diagnostic accuracy [24]; there is growing concern among experts that the clinical utility these diagnostic criteria may be seriously limited [25]. Several major problems have been highlighted in the literature. First, a high proportion of diagnoses of mental disorders are recorded as “unspecified” (the term used in ICD) or “not specified elsewhere” (the corresponding term in the DSM). This suggests that health professionals find the current categories difficult to use or imprecise for describing their patients, or do not find the nuances introduced by the diagnostic subtypes useful in their clinical practice. Second, a high proportion of people with mental health problems meet the criteria for two or more disorders [26].

Other studies have also reported problems with the diagnosis. The Netherlands Mental Health Survey and Incidence Study found that in around half of patients treated with antidepressants the diagnosis was not recorded [13]. Furthermore, between 10 % and 15 % of long-term continuous treatments with antidepressants were found to be no longer necessary. Conceivably, some patients may not have received a specific diagnosis because of the difficulty of coding certain poorly defined conditions in a situation in which the pressure on health staff is intense [27].

Thirdly, very often the same psychological or pharmacological treatment is effective for several different mental disorders [28]. One of the reasons for the limited clinical utility of current diagnostic systems is their extraordinary complexity and the inclusion, with each new review, of a greater number of categories with increasingly fine distinctions [26].

The most frequently prescribed drug during the early period (years 2003–2008) was paroxetine, followed by fluoxetine and sertraline. The most common prescription in the second period (2008–2011) was paroxetine, followed by escitalopram and then venlafaxine. A study conducted in Italy between 2000 and 2011 recorded an increase in the consumption of antidepressants and especially the group of selective serotonin reuptake inhibitors (SSRIs) [29].

The increase in the prescription of venlafaxine and escitalopram may reflect a policy of changing the active principle due to non-response to treatment. It is estimated that at least half of the people starting antidepressant treatment do not respond and a third remain depressed, despite the use of a variety of treatment strategies [21]. There has also been a change in prescription patterns on the part of physicians; a previous study reported an increase in the prescription of new molecules between 2002 and 2004 [30]. There is considerable commercial pressure on doctors to prescribe these new drugs, although this practice is not always justified by their clinical efficacy and safety.

Another reason for the increase in prescription of venlafaxine and escitalopram may be resistance to treatment. According to the clinical guidelines for depression, if treatment with SSRIs has proven ineffective, it can be replaced with venlafaxine, duloxetine or mirtazapine, and vice versa. If after a reasonable time no significant improvement is observed, an option would be to prescribe tricyclic antidepressants such as imipramine at doses of 150–300 mg/day [31].

Just over two-thirds of our patients (68.6 %) had received 1 or 2 antidepressant drugs at different times during the study period. The rest had consumed between three and thirteen different antidepressant drugs.

Overall compliance was 22 %. The compliance rate was 28 % in patients diagnosed with depression, and 21 % in those diagnosed with anxiety. The factors associated with increased treatment compliance were polypharmacy and a diagnosis of depressive or mixed disorder. The low overall rate of compliance may be associated with recurrences. It is known that recurrence risk in major depression is high; 50 % of patients have a new episode after the first one, 70 % after two, and as many as 90 % after three [32]. For this reason, an important question in the treatment of major depression is how long drug treatment should be maintained after recovery in order to prevent recurrence. Few studies have been specifically designed to address this issue and there is no clear consensus in the recommendations in other guidelines. In general, patients who abandon antidepressant treatment have a higher risk of recurrence than those who continue and, theoretically, patients with higher risk of recurrence would be the ones that would benefit the most from a prolonged treatment regimen [33]. Furthermore, the more prolonged the treatment, the smaller the difference in the risk of recurrence between treated patients and controls; that is, the benefit of extending treatment decreases over time [34]. Adjusting the duration of treatment after recovery to the type of patient is a considerable challenge and must be evaluated on a case-to-case basis.

Higher rates of compliance at older ages have been reported in some studies [35] which record shorter treatment periods in younger patients and in people in situations of socio-economic deprivation. However, in our study at multivariate level, compliance showed no significant differences with regard to age.

Among the various drugs prescribed, the highest compliance rates were observed with clomipramine, mirtazapine, maprotiline, and venlafaxine, and the lowest with paroxetine and trazodone. Other studies reported higher compliance with treatment with venlafaxine and duloxetine than with SSRIs, although this may be attributed to differences in clinical or pharmacological profiles. Serotonin reuptake inhibitors are used in first-line treatment with antidepressants. However, norepinephrine reuptake inhibitors are used in patients in more complex situations (i.e., recurrences or comorbidity) or in more severe cases [36].

Among the limitations of the data collection, the possible loss of some prescriptions should be borne in mind, because the drugs may have been dispensed over the counter or with prescriptions made by doctors outside the social security system. However, it has been estimated that these prescriptions account for a low percentage of the total in the health region; therefore, given the public health system’s universal coverage [37] the results of the survey can be considered valid. Furthermore, studies of this kind based on routine data bases lack information on cultural and social factors and on patients’ opinions, which also have an important bearing on the analysis of compliance. Another limitation is the lack of clinical information on the patients without a recorded diagnosis justifying treatment with antidepressants, and the absence of data regarding the severity of depression and patient response to antidepressants. It should also be borne in mind that we selected patients with an initial prescription in 2003 and who were prescribed antidepressant medication in 2008; we do not have information regarding their continuity over the intervening period or the number of episodes. This point should be considered when evaluating the results.

## Conclusions

In conclusion, we describe a cohort of patients who received treatment with antidepressants during the study period on two or more occasions. Almost half of the patients had no recorded diagnosis; a quarter had anxiety disorder, and another quarter depression or mixed anxiety-depression disorder. Approximately 1 in 4 patients complied with the treatment prescribed. The factors associated with compliance were polypharmacy and a diagnosis of depressive or mixed disorder. Drugs associated with increased compliance were clomipramine, mirtazapine, maprotiline and venlafaxine, while lower compliance was observed with paroxetine and trazodone after adjusting for different variables.

Access to diagnostic guidelines that are better suited to primary care would facilitate the compilation of patients’ clinical records and their treatments. Moreover, to improve compliance a deeper understanding of effective interventions is required, since abandonment is associated with an increase in the number of recurrences. Further studies are needed to define the treatment periods required in chronic or recurrent situations in the light of the patient’s symptoms and to identify the treatment durations associated with the fewest relapses. Progress in these areas would help to improve our approaches to these mental illnesses which have such a high prevalence in the population.
